# Impact of Li Decoration on P‑C_3_N Monolayer for Reversible Hydrogen Storage: A First-Principles Study

**DOI:** 10.1021/acs.langmuir.6c03071

**Published:** 2026-08-06

**Authors:** Amit Ramchiary, José A. S. Laranjeira, Luiz A. Ribeiro Jr, Julio R. Sambrano, Paritosh Mondal

**Affiliations:** † Department of Chemistry, 28686 Assam University, Silchar, Assam 788011, India; ‡ Modeling and Molecular Simulation Group, School of Sciences, 153994 São Paulo State University (UNESP), Bauru, São Paulo 17033-360, Brazil; § Institute of Physics, 28127 University of Brasília, Brasília, Federal District 70910-900, Brazil; ∥ Computational Materials Laboratory, LCCMat, Institute of Physics, University of Brasília, Brasília, Federal District 70910-900, Brazil

## Abstract

Two-dimensional carbon nitride materials have emerged as promising candidates for multifunctional applications. Motivated by the growing interest in carbon nitride-based two-dimensional materials, we investigated the hydrogen storage capacity of a Li-decorated P–C_3_N (Li@P–C_3_N) monolayer using density functional theory (DFT) calculations. Upon functionalization with Li atoms, the P–C_3_N monolayer can accommodate up to 12 H_2_ molecules, achieving a hydrogen storage capacity of 9.59 wt % with adsorption energies ranging from −0.18 to −0.13 eV per H_2_. The calculated material-level gravimetric capacity exceeds the numerical value of the DOE system-level gravimetric target. The hydrogen adsorption mechanism on Li@P–C_3_N was analyzed through charge transfer analysis, projected density of states (PDOS), and noncovalent interaction (NCI) analysis. The thermal stability of both pristine P–C_3_N and Li@P–C_3_N systems was examined using *ab initi* *o* molecular dynamics (AIMD). Furthermore, the dynamical stability of Li@P–C_3_N was confirmed through phonon dispersion calculations. The reversibility of hydrogen adsorption in the Li@P–C_3_N + 12H_2_ system was also investigated using AIMD simulations. These results demonstrate that Li-decorated P–C_3_N is a highly efficient candidate for hydrogen storage. Therefore, experimental investigations of P–C_3_N are strongly encouraged to validate its potential for reversible hydrogen storage applications.

## Graphical abstract

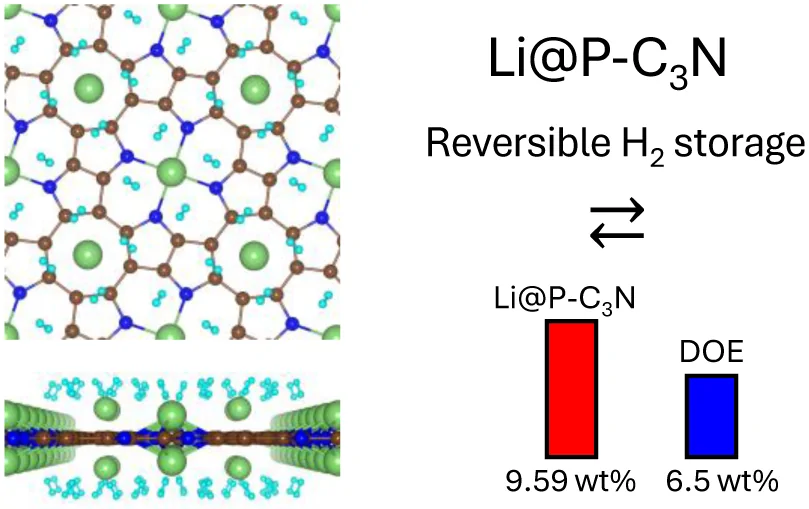

## Introduction

Due to rapid population growth and industrial development, global energy consumption is projected to increase by approximately 50% by 2040. Currently, most energy demands are met by fossil fuels, which significantly contribute to greenhouse gas emissions.^1,2^ Consequently, there is an urgent global need to transition from fossil-based energy systems to sustainable and environmentally friendly alternatives. In this context, hydrogen has emerged as a promising clean energy carrier due to its high abundance, renewability, zero-emission byproduct, and high gravimetric energy density of 120 MJ kg^–1^ (approximately three times that of gasoline, 44 MJ kg^–1^).^3,4^ Despite these advantages, the primary challenges in establishing a hydrogen economy remain the development of compact, safe, efficient, and cost-effective storage materials capable of operating under near-ambient conditions.^5^ Hydrogen storage technologies are generally categorized into physical-based and material-based systems. Physical-based storage includes compressed gas, cryo-compressed hydrogen, and liquid H_2_.^6^ However, these conventional approaches suffer from safety concerns, energy-intensive compression requirements, boil-off losses, and practical limitations for fuel cell electric vehicles (FCEVs).^7,8^ These limitations motivate the exploration of innovative material-based strategies for safe and efficient hydrogen storage.^3,9,10^

In contrast, material-based solid-state hydrogen storage offers distinct advantages, including higher storage capacity per unit mass, enhanced safety, and favorable adsorption/desorption kinetics through reversible interactions.^11^ The U.S. Department of Energy (DOE) has established system-level gravimetric targets for onboard reversible hydrogen storage. In addition, adsorption energies within an intermediate range are generally considered desirable in theoretical studies because they may provide a suitable balance between hydrogen retention and release, with values of approximately −0.10 to −0.60 eV per H_2  ​_ molecule commonly reported as favorable for reversible storage.^12^ −^13,14^ Meeting these criteria is essential for practical hydrogen storage applications.

Recently, two-dimensional (2D) materials have attracted significant attention for hydrogen storage applications due to their high surface area, tunable electronic properties, and structural stability.^15–18^ However, pristine 2D materials generally exhibit weak hydrogen adsorption energies that do not satisfy DOE targets. Various modification strategies have therefore been proposed to enhance hydrogen storage performance, including metal decoration,^19,20^ external electric field application,^21,22^ heteroatom doping,^23,24^ vacancy engineering,^25,26^ and strain engineering.^27,28^

Several recent studies have demonstrated promising hydrogen storage capacities in functionalized 2D systems. Beniwal et al. reported that Li doping in a holey biphenylene framework achieves 13.3 wt % hydrogen storage with adsorption energies ranging from −0.25 to −0.41 eV/H_2_.^29^ Tayyab et al. investigated Li- and Na-decorated Sc_3_N_2_ MXene monolayers and reported capacities of 5.9 and 5.6 wt %, respectively.^30^ Huzaifa et al. demonstrated that Li-decorated h-B_2_S_3_ nanosheets provide 6.35 wt % hydrogen storage with an average adsorption energy of −0.16 eV/H_2_.^31^ Similarly, Zhang et al. reported that CLi_3_-decorated C_3_N monolayers achieve 7.23 wt % with adsorption energies around −0.21 eV/H_2_.^32^ Othman et al. studied Sc-, Ti-, and V-functionalized C_3_N_5_ monolayers and reported hydrogen storage capacities of 9.65, 9.48, and 9.32 wt %, respectively.^33^ Martins et al. showed that Li- and Na-decorated inorganic graphenylene SiC yield 8.27 and 6.78 wt %, respectively.^34^ Rehman et al. further demonstrated that Li-decorated two-dimensional aluminum phosphide monolayers achieve 6.54 wt % with an average adsorption energy of −0.228 eV/H_2_.^35^

Among 2D materials, carbon nitrides stand out due to their nontoxicity, low cost, high stability, abundant active sites, and tunable electronic properties.^36,37^ Duan et al. reported reversible hydrogen storage on Mg-decorated C_7_N_3_ with 6.45 wt % capacity.^38^ Li-decorated graphitic-CN monolayers were shown to achieve 10.81 wt %.^39^ Chen et al. also demonstrated 5.7 wt % storage in Li-decorated α-C_3_N_2_.^40^ Tan et al. introduced new tetragonal carbon nitride monolayers, including P–C_3_N, confirming their structural stability.^41^ More recently, Na-functionalized T-C_3_N achieved 10.63 wt % hydrogen storage.^42^

Motivated by these findings, the present work employs density functional theory (DFT) calculations to investigate Li-decorated P–C_3_N as a potential reversible hydrogen storage material. Li atoms were selected due to their low atomic mass and strong polarization capability arising from their highly electropositive nature, which promotes enhanced hydrogen adsorption. Thermal and dynamical stability were evaluated using *ab initio* molecular dynamics (AIMD) simulations and phonon dispersion calculations. The adsorption mechanism was further analyzed using projected density of states (PDOS), charge density difference (CDD), Bader charge analysis, and noncovalent interaction (NCI) index calculations. Additionally, key performance metrics, including desorption temperature, metal diffusion barrier, and hydrogen occupation number, were systematically examined to comprehensively evaluate the hydrogen storage capability of the system.

## Computational Details

To study the hydrogen storage properties of the P–C_3_N monolayer, first-principles calculations within the framework of density functional theory (DFT) were performed using the Vienna *ab initio* simulation package (VASP).^43,44^ The exchange-correlation interactions were treated using the generalized gradient approximation (GGA) in the Perdew–Burke–Ernzerhof (PBE) formulation, to understand the electronic and geometric properties.^45^ The projected augmented-wave (PAW)^46^ pseudopotential was used to describe the core–valence interactions, along with a plane-wave basis at an energy cutoff of 520 eV for all calculations. To account for long-range van der Waals (vdW) interactions, the Grimme DFT-D4 ^47^ method is employed, which provides an empirical dispersion correction to enhance the accuracy of weak intermolecular interactions in the study of weak adsorption modeling. A vacuum spacing of 15 Å was applied perpendicular to the monolayer to avoid spurious interactions between periodic images. The Monkhorst–Pack^48^ *k*-point grid of 5 × 5 × 1 was employed to sample the Brillouin zone. All structures were completely relaxed using the conjugate gradient algorithm until the forces on each atom in the system were reduced to below 0.01 eV/Å, and the electronic self-consistency loop converged once the energy change between steps was less than 1 × 10^–5^ eV. *Ab initio* molecular dynamics (AIMD) simulations were conducted within the NVT ensemble using the Nosé thermostat^49^. The pristine P–C_3_N monolayer was simulated at 300 K for 5 ps, whereas Li@P–C_3_N and Li@P–C_3_N+12H_2_ were simulated for 20 ps at 450 and 300 K, respectively. A time step of 0.5 fs was used in all AIMD simulations.

Further, noncovalent interactions (NCI) are computed using the Multiwfn code in the studied materials.^50^

Hydrogen molecules were subsequently adsorbed near each of the Li atoms, and to evaluate the interaction of hydrogen with Lithium decorated P-*C*_3_N (Li@P-*C*_3_N), the adsorption energy per H_2_ is calculated using the equation:^51,52^

1 $$E_{\mathrm{ads}}=\frac{E_{\mathrm{Li}@P-C_{3}N+{\mathrm{nH}}_{2}}-E_{\mathrm{Li}@P-C_{3}N}-nE_{H_{2}}}{n}$$

where $E_{\mathrm{Li}@P-C_{3}N+{\mathrm{nH}}_{2}}$ is the total energy of the system with *n* adsorbed hydrogen molecules. $E_{\mathrm{Li}@P-C_{3}N}$ refers to the energy of the Li-decorated P-*C*_3_N system, while $E_{H_{2}}$ represents the energy of an isolated H_2_, respectively. The n indicates the number of H_2_ molecules present in the system.

In addition to the *E*_ads_, the stepwise adsorption energy (*E*_step_) was calculated to evaluate the energetic contribution of each successive H_2_ loading step. This descriptor allows us to determine whether the adsorption of additional H_2_ molecules remains favorable as the surface approaches saturation. It is defined as

2 $$E_{\mathrm{step}}=\frac{E_{\mathrm{Li}@P-C_{3}N+nH_{2}}-E_{\mathrm{Li}@P-C_{3}N+\left(n-m\right)H_{2}}-mE_{H_{2}}}{m}$$

where *m* is the number of H_2_ molecules added at each step. Here, *m* = 2, corresponding to the sequential addition of two H_2_ molecules around the Li adsorption centers. Therefore, *E*_step_ represents the incremental adsorption energy per H_2_ molecule for each loading stage. Negative values indicate that the additional H_2_ adsorption step is exothermic and thermodynamically favorable.

To check the anchoring strength of lithium atoms on P-*C*_3_N monolayer, the binding energy (*E*_*b*_) of Li was calculated using the standard formulation:^53,54^

3 $$E_{b}=\frac{E_{\mathrm{Li}@P-C_{3}N}-E_{P-C_{3}N}-mE_{\mathrm{Li}}}{m}$$

where $E_{\mathrm{Li}@P-C_{3}N}$ and $E_{P-C_{3}N}$ are the total energies of the Li-decorated and pristine P-C_3_N systems, respectively, and *E*_Li_is the total energy of an isolated Li atom, and *m* is the number of Li atoms per unit cell.

The Bader scheme^55^ was applied to perform charge transfer analyses, and the charge density difference (CDD) plots were generated to visualize the redistribution of electrons. The CDD for Li@P-*C*_3_N was calculated using the equation:

4 $$\Delta \rho =\rho_{\mathrm{mLi}@P-C_{3}N}-\rho_{P-C_{3}N}-\rho_{\mathrm{mLi}}$$

where *ρ*_mLi@P–C3N_ is the charge density of the Li-decorated P–C_3_N, *ρ*_P–C3N_ represents the charge density of the substrate P-C_3_N, while ρ_mLi_ denotes the charge density of Li.

However, for the hydrogen adsorption system, the CDD plot is evaluated using the equation:

5 $$\Delta \rho =\rho_{\mathrm{Li}@P-C_{3}N+nH_{2}}-\rho_{\mathrm{Li}@P-C_{3}N}-\rho_{{\mathrm{nH}}_{2}}$$

where *ρ*Li@P–C_3_N+*nH*_2_ represents the charge density of the hydrogen adsorbed on the Li-decorated P-*C*_3_N system,*ρ*Li@P–C_3_N is the charge density of the Li@P-C_3_N system, while *ρ*nH_2_ denotes the charge densities of the n isolated H_2_ molecules, calculated at the same positions and geometries as in the combined system.

The hydrogen storage capacity (HSC) in gravimetric weight percentage of the Li@P-*C*_3_N+*n*H_2_ was calculated using the equation:

6 $$\mathrm{HSC}\left(\mathrm{wt}\%\right)=\left(\frac{nM_{H_{2}}}{nM_{H_{2}}+M_{\mathrm{mLi}@P-C_{3}N}}\right)\times 100$$

where $nM_{H_{2}}$ is the total mass of the *n*H_2_ molecules, and $M_{\mathrm{mLi}@P-C_{3}N}$ is the mass of Li-decorated P-C_3_N system.

The desorption temperature (*T*_*D*_) is calculated using the van’t Hoff relation:^56–58^

$$T_{D}=\frac{\left|E_{\mathrm{ads}}\right|}{k_{B}\left(\Delta S/R-\ln\left(P/P_{0}\right)\right)}$$ 7

where *k*_*B*_ is the Boltzmann constant (1.38 × 10^–23^ J K^–1^), Δ*S* is the magnitude of the change in entropy of hydrogen from gas to liquid phase (75.44 J mol^–1^ K^–1^), *R* is the universal gas constant (8.314 J mol^–1^ K^–1^) and P is the equilibrium pressure. Before substitution into eq 7, the adsorption energies calculated in eV per H_2_ molecule were converted to joules per molecule.

Finally, the practical applicability of the H_2_ storage material was assessed using a finite-state grand-canonical thermodynamic model. In this approach, the adsorbed phase is represented by a discrete set of sequential adsorption states containing *n* = 0,···,*N*_max_ H_2_ molecules, while the gas phase acts as a molecular reservoir controlled by temperature and pressure. Therefore, the number of adsorbed H_2_ molecules is allowed to fluctuate, which is consistent with a grand-canonical description.

The incremental adsorption energies obtained from DFT, *E*_inc_(*i*), were used to construct the cumulative adsorption enthalpy for each loading state:

8 $$\Delta H_{n}=\overset{n}{\underset{i=1}{\sum }}E_{\mathrm{inc}}\left(i\right)$$

where Δ*H*_0_ = 0. The DFT energies were converted from eV per H_2_ molecule to J mol^–1^ in order to maintain consistency with the gas constant *R*. The corresponding temperature-dependent free energy was then estimated as

9 $$\Delta G_{n}\left(T\right)=\Delta H_{n}-nT\Delta S_{\mathrm{ads}}$$

where Δ*S*_ads_ is the effective entropy change associated with the adsorption of one H_2_ molecule. In the present model, Δ*S*_ads_ was taken as −75.44 J mol^–1^ K^–1^, so that the term −*nT*Δ*S*_ads_ represents the entropic penalty associated with transferring H_2_ from the gas phase to the adsorbed state.

The pressure dependence was introduced through the chemical potential of the H_2_ reservoir, assuming ideal-gas behavior:

10 $$\mu \left(T,P\right)-\mu^{◦}\left(T\right)=RT\ln\left(\frac{P}{P_{0}}\right)$$

where *P*_0_ = 1 atm is the reference pressure. Thus, the statistical weight of each adsorption state was calculated as

11 $$w_{n}=\exp\left[-\frac{\Delta G_{n}\left(T\right)-nRT\ln\left(P/P_{0}\right)}{RT}\right]$$

The discrete grand-canonical partition function is then given by

12 $$Z=\overset{N_{\max}}{\underset{n=0}{\sum }}w_{n}$$

and the mean number of adsorbed hydrogen molecules is obtained from

13 $$\left\langle N\right\rangle =\frac{\overset{N_{\max}}{\underset{n=0}{\sum }}nw_{n}}{Z}$$

Finally, the occupation index was defined as

14 $$\theta =\frac{\left\langle N\right\rangle }{N_{\max}}$$

where θ = 0 corresponds to the empty surface and θ = 1 corresponds to full occupation within the set of adsorption states considered.

This model was used to construct temperature–pressure occupation maps in the range of 100–600 K and 1–60 atm. In addition, one-dimensional cuts at selected pressures were extracted to evaluate the temperature dependence of both θ and ⟨*N*⟩. The DOE-relevant temperature interval was highlighted in these plots to facilitate the assessment of the H_2_ loading behavior under near-ambient operating conditions.

## Results and Discussion

### Structural Stability and Electronic Properties of Pristine P–C_3_N

Prior to investigating Li decoration and hydrogen adsorption, the structural stability and intrinsic electronic properties of the pristine P–C_3_N monolayer were systematically examined. The optimized top and side views of the pristine penta-C_3_N (P–C_3_N) monolayer are presented in Figure 1a. Structurally, the system consists of a two-dimensional carbon nitride framework composed of interconnected penta–octagonal rings with a stoichiometric C:N ratio of 3:1. The optimized geometry belongs to the tetragonal space group *P*4/*m* (No. 83) with a lattice constant of 7.03 Å. Six distinct bond lengths are identified in the optimized structure, namely d1 = 1.45 Å (C–C), d2 = 1.32 Å (C–N), d3 = 1.40 Å (C–N), d4 = 1.39 Å (C–C), d5 = 1.48 Å (C–C), and d6 = 1.45 Å (C–C), as illustrated in Figure 1a. Based on the surface symmetry and local coordination environment, the potential adsorption sites were categorized into hollow sites (H1–H3), bridge sites (B1–B4), and top sites (T1–T3), which are highlighted in violet, green, and cyan colors, respectively.

**Figure 1:**
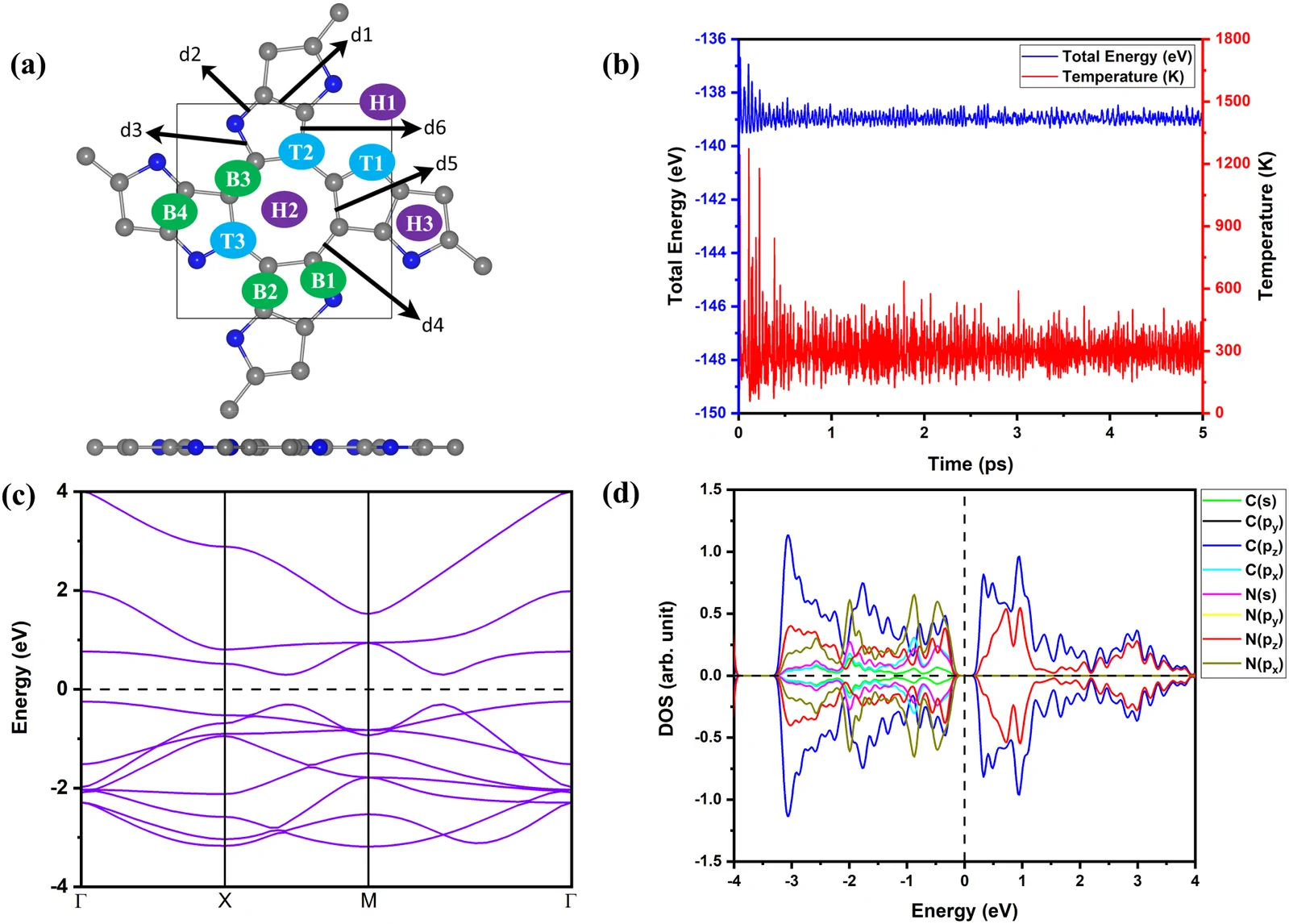
(a) Top and side views of the relaxed pristine P–C_3_N monolayer. The adsorption sites are classified as top sites (T1–T3), bridge sites (B1–B4), and hollow sites (H1–H3). Carbon and nitrogen atoms are represented in gray and blue spheres. (b) The total energy and temperature profile from the AIMD simulation at 300 K for 5 ps duration. (c) Electronic band structure of pristine P-C_3_N, and (d) Projected density of states (PDOS) of P-C_3_N for separate orbital contribution of C and N atoms, respectively.

To further evaluate the thermal stability of the pristine monolayer, *ab initio* molecular dynamics (AIMD) simulations were performed at 300 K for 5 ps using a time step of 0.5 fs. As shown in Figure 1b, both the total energy and temperature exhibit only small fluctuations throughout the simulation, indicating stable thermal equilibration under ambient conditions. Moreover, no noticeable structural distortion or bond breaking is observed in the final AIMD snapshot after 5 ps (Figure S1), confirming the thermal stability of the P–C_3_N monolayer.

The electronic properties of pristine P–C_3_N were subsequently investigated through band structure and projected density of states (PDOS) analyses. The electronic band structure shown in Figure 1c reveals that the monolayer is a semiconductor with an indirect band gap of 0.54 eV at the GGA–PBE level, in good agreement with previous theoretical reports.^41^ The relatively narrow band gap suggests favorable electronic conductivity and potential tunability through chemical functionalization or metal decoration. Furthermore, the electronic bands near the Fermi level exhibit relatively dispersive characteristics along the X → M and M → Γ high-symmetry directions, implying enhanced carrier mobility within the two-dimensional framework.

Further insight into the electronic structure is obtained from the PDOS analysis presented in Figure 1d. The valence band region is predominantly governed by C­(*p*_*z*_) states, together with noticeable contributions from N­(*s*), N­(*p*_*z*_), and N­(*p*_*x*_) orbitals, whereas the contributions from C­(*s*) and C­(*p*_*x*_) orbitals remain comparatively weak. Near the valence band maximum (VBM), the dominant contribution arises from N­(*p*_*x*_) states. In contrast, the conduction band minimum (CBM) mainly originates from hybridized C­(*p*_*z*_) and N­(*p*_*x*_) orbitals, indicating the presence of delocalized π-type electronic states across the monolayer. In addition, the symmetric distribution of spin-up and spin-down states around the Fermi level confirms the nonmagnetic nature of pristine P–C_3_N.

After establishing the structural and electronic stability of pristine P–C_3_N, the adsorption behavior of a single H_2_ molecule was systematically investigated at the various adsorption sites shown in Figure 1a. The corresponding hydrogen adsorption energies (*E*_ads_) were calculated using eq 1. As summarized in Table 1, most adsorption sites exhibit weak interactions, with adsorption-energy magnitudes below 0.11 eV per H_2_ molecule, suggesting weak physisorption and indicating that pristine P–C_3_N alone is insufficient for efficient reversible hydrogen storage. Among the considered sites, only the H1 configuration exhibits a comparatively stronger interaction, with an adsorption energy of −0.11 eV per H_2_. Nevertheless, this interaction strength remains inadequate to achieve the target gravimetric hydrogen storage capacity of 6.5 wt %.

**Table 1:** Adsorption Energies (*E*_ads_) of a Single H_2_ Molecule on Different Adsorption Sites of the P–C_3_N Monolayer

Initial Site	Final Site	*E*_ads_ (eV/H_2_)
B1	B1	–0.06
B2	B2	–0.06
B3	B3	–0.06
B4	B4	–0.06
H1	H1	–0.11
H2	H2	–0.06
H3	H3	–0.06
T1	H1	–0.11
T2	T2	–0.06
T3	T3	–0.06

The optimized adsorption geometries for all considered configurations are displayed in Figure S2. The equilibrium distance between the adsorbed H_2_ molecule and the P–C_3_N surface lies within the range of 2.81–3.31 Å, further confirming the physisorptive nature of hydrogen adsorption on the pristine monolayer. Overall, these findings demonstrate that pristine P–C_3_N is not an efficient hydrogen storage material in its native form. Therefore, appropriate surface modification strategies, such as alkali metal decoration, are required to enhance the adsorption strength and improve the hydrogen storage performance of the system.

### Interaction of Lithium and P–C_3_N

To evaluate the adsorption stability of Li atoms on the P–C_3_N monolayer, the binding energies of Li at different adsorption sites, including top (T1–T3), bridge (B1–B4), and hollow (H1–H3) positions, were calculated using eq 3. The optimized geometries reveal that Li atoms initially placed at top and bridge sites spontaneously migrate toward neighboring hollow regions during structural relaxation, indicating that hollow sites are energetically more favorable for Li adsorption. Among the considered configurations, the H1 and H2 hollow sites exhibit the strongest adsorption tendencies and are therefore identified as the most stable adsorption sites for Li decoration. The optimized configurations of Li adsorption on both sides of the P–C_3_N monolayer are shown in Figure 2a.

**Figure 2:**
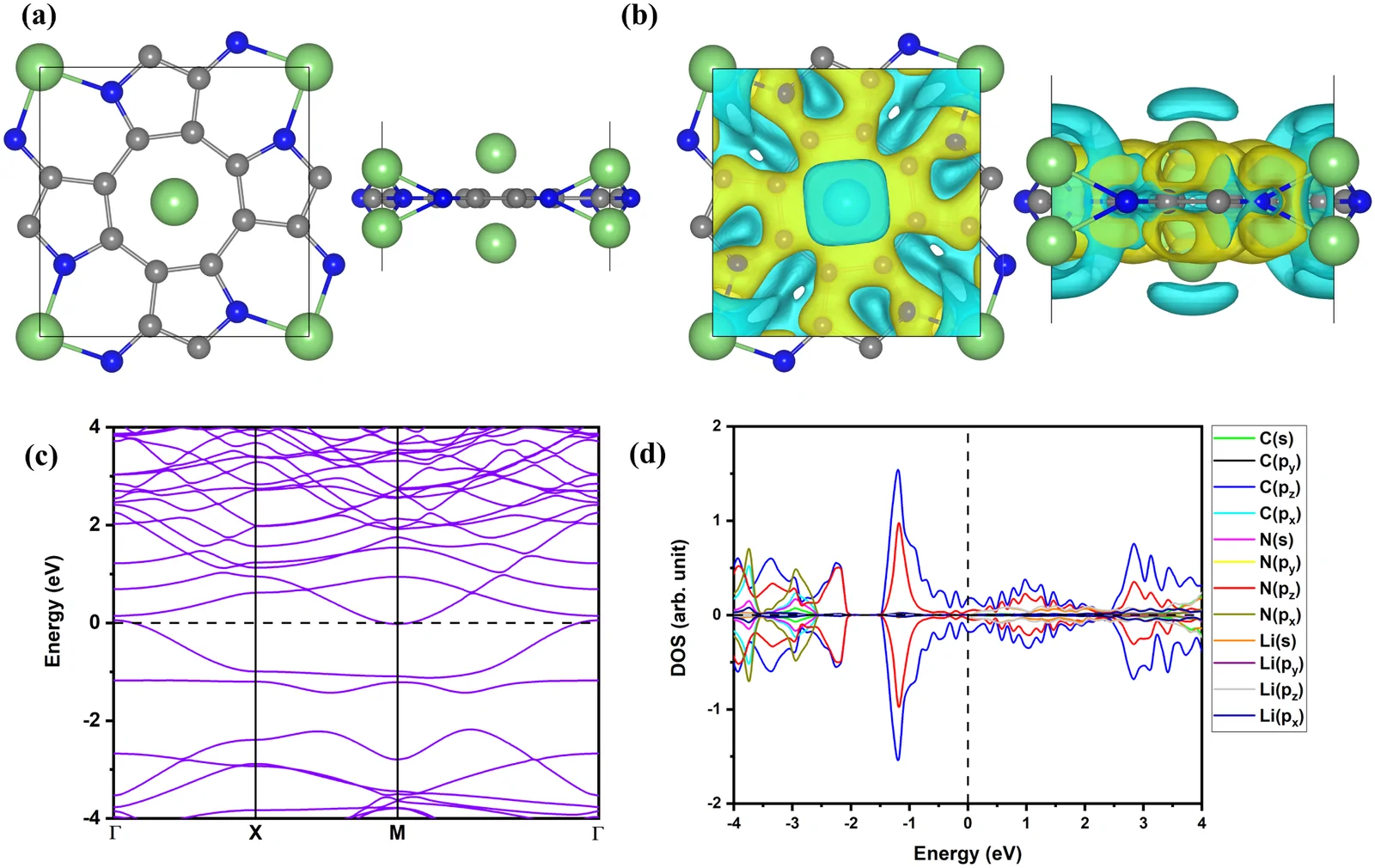
(a) Top and side views of the optimized Li-decorated P–C_3_N monolayer, where Li atoms are shown in light green color. (b) Charge density difference (CDD) isosurface of Li@P–C_3_N plotted at 0.001 e/Å ^3^, where yellow and cyan regions represent charge accumulation and charge depletion, respectively. (c) Electronic band structure and (d) projected density of states (PDOS) of Li@P–C_3_N, demonstrating the semiconductor-to-metal transition induced by Li decoration and the orbital contributions near the Fermi level.

The calculated binding energy of Li is approximately −3.03 eV per Li atom, indicating strong interaction between Li adatoms and the P–C_3_N surface. Importantly, this value is substantially larger than the cohesive energy of bulk Li metal, suggesting that Li aggregation is energetically unfavorable and that isolated Li adsorption can be maintained on the monolayer surface. Structurally, each Li atom adsorbed at the H1 site is coordinated with four neighboring N atoms, whereas Li adsorption at the H2 site is stabilized by interaction with the surrounding octagonal carbon ring. The equilibrium vertical distances between Li and the monolayer plane are calculated to be 0.92 and 1.39 Å for the H1 and H2 sites, respectively. The shorter adsorption height at the H1 site indicates comparatively stronger interaction with the substrate, which can be attributed to the enhanced electrostatic interaction between electropositive Li atoms and electronegative N atoms in the P–C_3_N framework. Furthermore, Li adsorption at the H1 and H2 hollow sites enables a maximum loading of four Li atoms per P–C_3_N unit cell without inducing noticeable structural distortion. The relatively large separations between adjacent Li atoms (4.99 and 7.03 Å) effectively suppress metal clustering and ensure uniform dispersion of Li adatoms across the surface, thereby providing an energetically and structurally favorable platform for efficient hydrogen storage applications.

To further investigate the interaction mechanism between Li atoms and the P–C_3_N monolayer, charge density difference (CDD) analysis was performed using eq 4. The resulting CDD isosurfaces at 0.001 e/Å ^3^ are illustrated in Figure 2b, where cyan and yellow regions represent charge depletion and accumulation, respectively. Pronounced charge depletion is observed around the Li adatoms, whereas substantial charge accumulation appears on the neighboring regions of the P–C_3_N framework. This charge redistribution confirms electron donation from Li atoms to the monolayer. The Bader charge analysis further supports the strong electronic interaction between the Li adatoms and the P–C_3_N monolayer. A site-dependent charge donation is observed: the Li atoms adsorbed on the H2 sites, located above the octagonal carbon rings, exhibit a positive Bader charge of approximately +0.87 *e* each, whereas the Li atoms adsorbed on the H1 sites, coordinated by the surrounding N atoms, exhibit a positive Bader charge of approximately +0.74 *e* each. Thus, the Li adatoms act as electron donors, transferring charge to the P–C_3_N substrate and producing positively charged adsorption centers on the surface. The slightly larger charge depletion of Li at the H2 site suggests a stronger charge-transfer interaction with the carbon-rich octagonal cavity, while the N-coordinated H1 site also provides effective electrostatic stabilization. This charge redistribution is crucial for preventing Li aggregation and for enhancing the affinity of the decorated monolayer toward H_2_ adsorption, since the positively charged Li centers can polarize incoming H_2_ molecules and promote reversible physisorption.

The electronic properties of Li-decorated P–C_3_N were further examined through band structure and projected density of states (PDOS) analyses, as presented in Figure 2c,d. In contrast to pristine P–C_3_N, which exhibits semiconducting behavior, Li decoration induces metallic characteristics, as evidenced by the appearance of bands crossing the Fermi level in Figure 2c. This semiconductor-to-metal transition arises from charge transfer between Li adatoms and the substrate and is expected to facilitate electronic conductivity as well as improve interaction with adsorbed hydrogen molecules.

The PDOS analysis shown in Figure 2d indicates that the lower valence band region is mainly dominated by C­(*p*_*z*_) and N­(*p*_*z*_) orbitals, similar to the pristine system. However, significant modifications are observed near the Fermi level after Li adsorption. In particular, Li­(*s*) and Li­(*p*) orbitals contribute prominently around the Fermi energy, confirming the active participation of Li states in the electronic structure. Simultaneously, the reduced contribution from N­(*p*_*z*_) states near the Fermi level further supports the occurrence of electron donation from Li atoms to the substrate. Moreover, the enhanced contribution of C­(*p*_*z*_) states suggests that carbon atoms act as major charge-accepting centers, thereby contributing to the stabilization of Li adsorption and the metallic nature of the decorated system.

The thermal stability of the Li-decorated P–C_3_N system was investigated through *ab initio* molecular dynamics (AIMD) simulations at 450 K for 20 ps with a time step of 0.5 fs. The corresponding energy and temperature profiles are shown in Figure 3a. Throughout the simulation, both the total energy and temperature fluctuate within a relatively narrow range without any abrupt structural disruption, confirming the thermal stability of the Li-decorated system. Furthermore, the final AIMD configuration after 20 ps, shown in Figure S3, demonstrates that all Li atoms remain strongly anchored at their respective hollow adsorption sites without clustering or surface diffusion. During the simulation, the Li–surface distances exhibit only minor variations, changing from 0.98 to 1.38 Å for the H1 site and from 1.39 to 1.60 Å for the H2 site. These small fluctuations do not significantly affect the adsorption stability, further demonstrating the robustness of Li anchoring on the P–C_3_N surface under elevated-temperature conditions.

**Figure 3:**
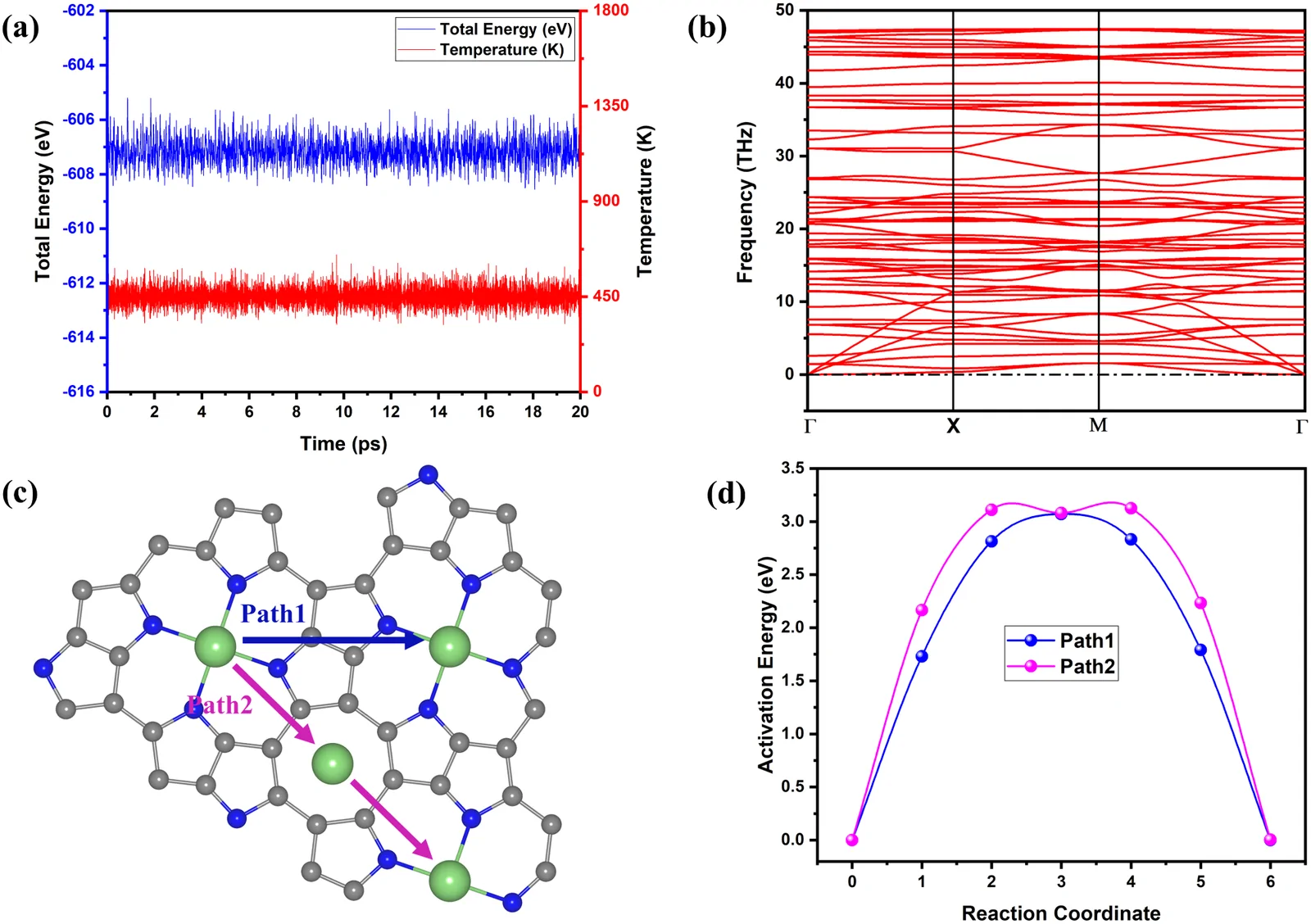
(a) Total energy and temperature fluctuations obtained from AIMD simulations of the Li@P–C_3_N system at 450 K for 20 ps, demonstrating the thermal stability of Li decoration. (b) Phonon dispersion spectrum of Li@P–C_3_N along the high-symmetry path Γ–X–M−Γ, confirming dynamical stability through the absence of imaginary frequencies. (c) Schematic illustration of the two possible Li diffusion pathways on the P–C_3_N monolayer considered for nudged elastic band (NEB) calculations. (d) Corresponding diffusion energy barrier profiles for Li migration along path 1 and path 2 obtained using the NEB method.

To examine the dynamical stability of the Li@P–C_3_N system, phonon dispersion calculations were carried out along the high-symmetry path Γ–X–M−Γ, and the results are displayed in Figure 3b. The absence of imaginary phonon frequencies confirms the dynamical stability of the Li-decorated monolayer. Therefore, both AIMD and phonon analyses consistently demonstrate that the Li@P–C_3_N system is thermally and dynamically stable, satisfying essential stability criteria for practical hydrogen storage applications.

Metal agglomeration is a major challenge in metal-decorated hydrogen storage materials because it reduces the number of active adsorption sites and deteriorates storage performance.^59^ To evaluate the tendency of Li diffusion and possible clustering on the P–C_3_N surface, nudged elastic band (NEB) calculations were performed along two representative diffusion pathways, as illustrated in Figure 3c.^60,61^

Before discussing the calculated barriers, it is important to clarify the construction of the NEB models. For the adsorption of a single Li atom on P–C_3_N, the H1 site is the most favorable adsorption site, with Li tending to relax toward the plane of the monolayer. However, in the optimized configuration containing two Li atoms, the Li adatoms are stabilized on opposite sides of the sheet, one above and one below the P–C_3_N plane, with a Li–surface distance of approximately 0.98 Å at the H1 site. Since this out-of-plane configuration is more representative of the Li-decorated system used for H_2_ adsorption, the NEB calculations were designed to isolate the lateral migration of a single Li adatom while preserving the relevant adsorption geometry of the decorated structure. Therefore, the Li–surface distance obtained from the optimized 2Li@P–C_3_N configuration was used as a reference to construct the initial diffusion pathway for one individual Li atom along paths 1 and 2. This procedure avoids mixing the in-plane relaxation tendency of an isolated Li atom with the lateral diffusion process and allows the intrinsic migration barrier of an exposed Li adsorption center to be estimated under conditions closer to those of the decorated surface.

The corresponding diffusion energy profiles are shown in Figure 3d. The calculated diffusion barriers are 3.08 and 3.11 eV for path 1 and path 2, respectively. These values are considerably higher than those reported for several previously studied Li-decorated systems, including boron-doped graphene nanoribbons (0.56 eV), pristine graphene (0.28 eV), and para B–B pair-doped graphene (0.50 eV).^62,63^ The large and nearly equivalent barriers obtained for both migration pathways indicate that Li diffusion over the P–C_3_N surface is strongly kinetically hindered. This behavior effectively suppresses Li migration across the surface and strongly inhibits metal agglomeration. Consequently, the Li@P–C_3_N system exhibits excellent anchoring stability, which is highly desirable for maintaining uniformly distributed active Li sites during reversible hydrogen adsorption and desorption processes.

### Hydrogen Storage Capacity of Li-Decorated P–C_3_N

To evaluate the hydrogen adsorption capability of the Li-decorated P–C_3_N monolayer, H_2_ molecules were sequentially introduced onto the Li@P–C_3_N surface at a loading of two H_2_ molecules per adsorption step, followed by full structural relaxation. The optimized adsorption configurations are illustrated in Figure 4. The average hydrogen adsorption energies (*E*_ads_), calculated using eq 1, together with the corresponding stepwise adsorption energies (*E*_step_), H–H bond lengths, Li–H distances, desorption temperatures (*T*_*D*_), and hydrogen storage capacities (HSC), are summarized in Table 2. The stepwise adsorption energy was included to evaluate the energetic favorability of each successive hydrogen-loading stage and to identify the saturation regime of the Li@P–C_3_N surface.

**Table 2:** Average Adsorption Energy (*E*_ads_), Stepwise Adsorption Energy (*E*_step_), H–H Bond Length and Li–H Distances, Desorption Temperature (*T_D_*), and Hydrogen Storage Capacity (HSC) for Li@P–C_3_N with Different Numbers of Hydrogen Molecules

System	*E*_ads_ (eV/H_2_)	*E*_step_ (eV/H_2_)	H–H (Å)	Li–H (Å)	*T*_*D*_ (K)	HSC (wt %)
Li@P–C_3_N+2H_2_	–0.13	–0.13	0.76	2.92	166.26	1.74
Li@P–C_3_N+4H_2_	–0.13	–0.13	0.76	2.66	166.26	3.42
Li@P–C_3_N+6H_2_	–0.18	–0.28	0.76	2.48	229.19	5.04
Li@P–C_3_N+8H_2_	–0.17	–0.14	0.76	2.17	215.30	6.61
Li@P–C_3_N+10H_2_	–0.15	–0.06	0.76	2.30	188.28	8.13
Li@P–C_3_N+12H_2_	–0.13	–0.04	0.75	2.40	166.26	9.59

**Figure 4:**
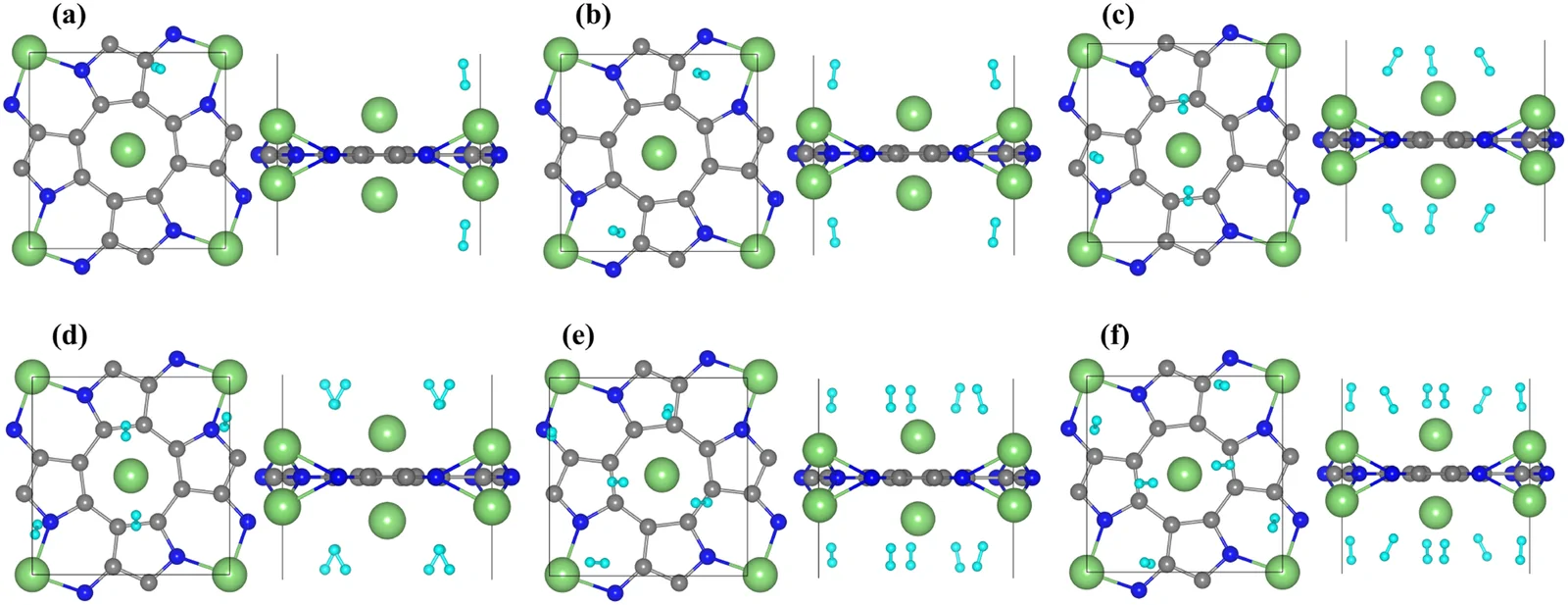
Optimized top and side views of Li@P–C_3_N upon sequential adsorption of 2 to 12 H_2_ molecules, (a) Li@P–C_3_N+2H_2_, (b) Li@P–C_3_N+4H_2_, (c) Li@P–C_3_N+6H_2_, (d) Li@P–C_3_N+8H_2_, (e) Li@P–C_3_N+10H_2_, and (f) Li@P–C_3_N+12H_2_. Hydrogen molecules (cyan) remain intact and symmetrically distributed around Li sites.

For the initial adsorption configuration, Li@P–C_3_N+2H_2_ (Figure 4a), the average adsorption energy is calculated to be −0.13 eV per H_2_. The corresponding stepwise adsorption energy is also −0.13 eV per H_2_, indicating that the first hydrogen-loading step is energetically favorable. The H–H bond length slightly elongates from 0.75 Å in an isolated H_2_ molecule to 0.76 Å, indicating weak perturbation of the molecular bond and suggesting molecular physisorption. The average Li–H distance is found to be 2.92 Å, reflecting weak electrostatic interaction between the polarized H_2_ molecules and positively charged Li centers. Upon increasing the hydrogen coverage to four H_2_ molecules (Li@P–C_3_N+4H_2_; Figure 4b), both *E*_ads_ and *E*_step_ remain equal to −0.13 eV per H_2_, while the H–H bond length remains nearly unchanged. Meanwhile, the Li–H distance decreases to 2.66 Å, indicating a slightly enhanced interaction with increasing hydrogen loading.

For the Li@P–C_3_N+6H_2_ configuration (Figure 4c), the average adsorption energy becomes more negative, reaching −0.18 eV per H_2_, which corresponds to the strongest average interaction among all investigated coverages. Notably, this configuration also exhibits the most favorable stepwise adsorption energy, with *E*_step_ = −0.28 eV per H_2_. This result indicates that the adsorption of the third H_2_ pair is particularly favorable, suggesting an efficient utilization of the Li-induced electrostatic adsorption sites. The associated H–H and Li–H distances are 0.76 and 2.48 Å, respectively, further supporting a molecular adsorption mechanism without H_2_ dissociation.

Further hydrogen adsorption leads to the formation of Li@P–C_3_N+8H_2_ and Li@P–C_3_N+10H_2_ systems (Figure 4d,e), with average adsorption energies of −0.17 and −0.15 eV per H_2_, respectively. The corresponding stepwise adsorption energies are −0.14 and −0.06 eV per H_2_, respectively. Although the magnitude of *E*_step_ decreases at higher coverages, the negative values indicate that the successive hydrogen adsorption process remains exothermic. This gradual weakening is expected as the available adsorption sites become progressively occupied and H_2_–H_2_ repulsive effects become more relevant. In both cases, the H–H bond length remains nearly constant at 0.76 Å, whereas the Li–H distances are 2.17 and 2.30 Å, respectively, suggesting effective polarization of H_2_ molecules around the Li adsorption centers.

At maximum loading, the Li@P–C_3_N monolayer can accommodate up to 12H_2_ molecules, corresponding to the Li@P–C_3_N+12H_2_ configuration shown in Figure 4f. The calculated average adsorption energy at this saturation coverage is −0.13 eV per H_2_, while the stepwise adsorption energy remains negative, *E*_step_ = −0.04 eV per H_2_. Although this value indicates weaker interaction for the final adsorption step, it still confirms that the complete hydrogenation process up to 12H_2_ is energetically favorable. The H–H and Li–H distances are 0.75 and 2.40 Å, respectively, confirming that the adsorbed hydrogen molecules preserve their molecular character. Beyond this loading, additional H_2_ molecules exhibit negligible interaction with the substrate, as evidenced by insignificant changes in H–H bond length, adsorption energies weaker than −0.10 eV per H_2_, and Li–H distances exceeding 3 Å. These results indicate that the adsorption saturation limit has been reached.

To further elucidate the interaction mechanism between hydrogen molecules and the Li@P–C_3_N system, charge density difference (CDD), Bader charge, band structure, and projected density of states (PDOS) analyses were performed. The top and side views of the CDD isosurfaces for Li@P–C_3_N+12H_2_, calculated using eq 5, are presented in Figure 5a,b. In these plots, yellow and cyan regions correspond to charge accumulation and charge depletion, respectively. Distinct charge redistribution regions are observed around both Li atoms and adsorbed H_2_ molecules. In particular, the simultaneous appearance of charge accumulation and depletion along the H–H bond axis indicates polarization of the H_2_ molecules induced by the electrostatic field generated by the positively charged Li adatoms.

**Figure 5:**
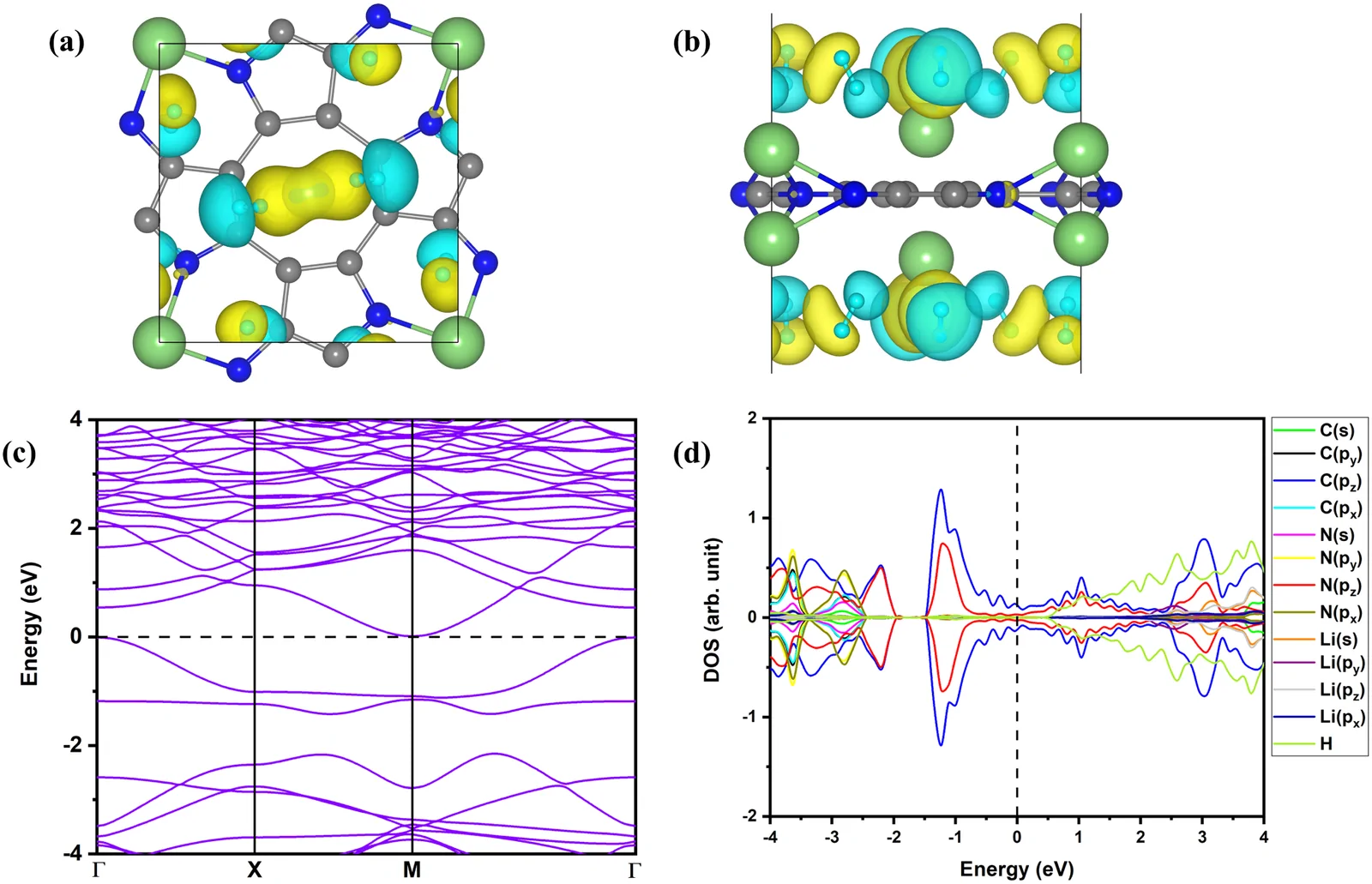
Charge density difference (CDD) plots of Li@P–C_3_N+12H_2_ shown from the top (a) and side (b) views at an isosurface value of 0.0009 e/Å^3^, where yellow and cyan regions represent charge accumulation and depletion, respectively. (c) Electronic band structure and (d) projected density of states (PDOS) of the Li@P–C_3_N+12H_2_ system, illustrating the metallic nature and the weak hybridization between adsorbed H_2_ molecules and the Li-decorated P–C_3_N monolayer.

The Bader charge analysis further confirms this interaction mechanism. The Li atoms adsorbed at the H1 and H2 sites retain positive charges of approximately +0.84 *e* and +0.85 *e*, respectively, even after hydrogen adsorption. Meanwhile, each adsorbed H_2_ molecule acquires a small amount of charge ranging from approximately −0.05 *e* to +0.11 *e*. This charge redistribution demonstrates that the interaction between H_2_ molecules and the Li@P–C_3_N substrate is primarily governed by electrostatic polarization rather than strong covalent bonding.

The electronic properties of the hydrogen-adsorbed system were further investigated through band structure and PDOS calculations. As shown in Figure 5c, the Li@P–C_3_N+12H_2_ system preserves the metallic character of Li@P–C_3_N, with several electronic bands crossing the Fermi level. This observation indicates that hydrogen adsorption does not significantly alter the electronic structure of the substrate and further supports the weak interaction between H_2_ molecules and the Li-decorated surface. The PDOS analysis presented in Figure 5d reveals that hydrogen-derived states contribute only minimally near the Fermi level, whereas more noticeable contributions appear in the higher conduction-band region. The weak hybridization between H orbitals and substrate electronic states confirms that hydrogen adsorption occurs predominantly through physisorption.

To gain further insight into the nature of these weak interactions, noncovalent interaction (NCI) analysis was carried out using the reduced density gradient (RDG) approach.^64^ The RDG function is defined as

15 $$s\left(r\right)=\frac{1}{2{\left(3\pi^{2}\right)}^{1/3}}\frac{\left|\nabla \rho \left(r\right)\right|}{\rho {\left(r\right)}^{4/3}}$$

where ∇ρ­(**r**) and ρ­(**r**) represent the gradient and magnitude of the electron density, respectively.

The RDG isosurfaces and corresponding scatter plots of *s*(**r**) versus sign­(λ_2_)­ρ before and after hydrogen adsorption are displayed in Figure 6. The color-coded isosurfaces distinguish different types of interactions: blue regions indicate strong attractive interactions, red regions correspond to steric repulsion, and green regions near sign­(λ_2_)­ρ ≈ 0 represent weak van der Waals interactions. As shown in Figure 6a,b, blue regions between Li atoms and the P–C_3_N monolayer confirm strong binding interactions that stabilize the decorated structure. In contrast, the red regions observed between adjacent Li atoms indicate repulsive interactions that suppress Li clustering and agglomeration.

**Figure 6:**
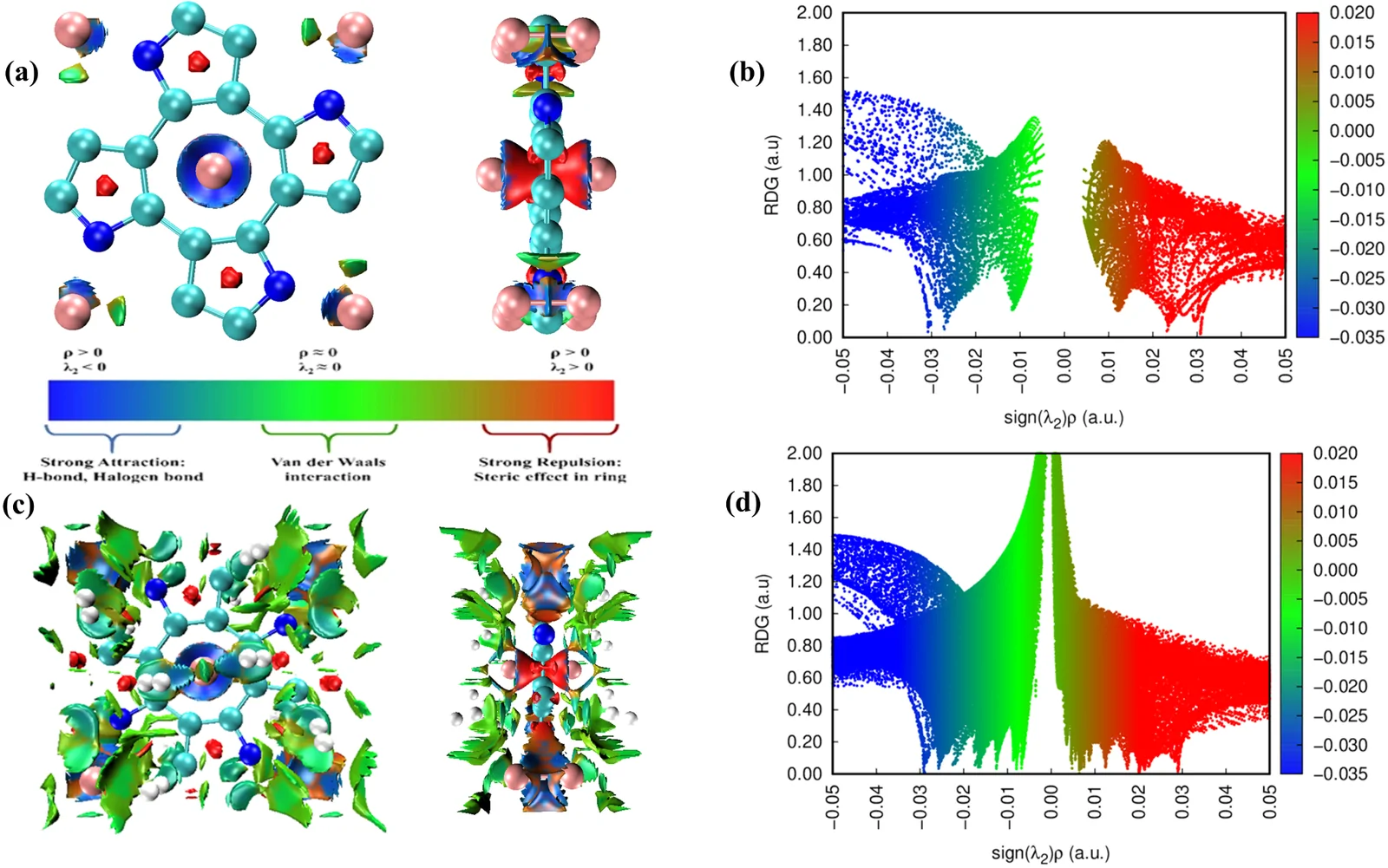
Reduced density gradient iso-surfaces and the corresponding NCI scatter plots of RDG vs sign (λ_2_)­ρ for (a, b) Li@P–C_3_N, and (c, d) Li@P–C_3_N+12H_2_, respectively.

After hydrogen adsorption, a substantial increase in green-colored regions is observed between the adsorbed H_2_ molecules and the Li@P–C_3_N surface, as illustrated in Figure 6c. This feature is further corroborated by the corresponding RDG scatter plot shown in Figure 6d, where a large concentration of data points appears near sign­(λ_2_)­ρ ≈ 0. The dominance of these green regions confirms that van der Waals interactions are primarily responsible for hydrogen adsorption. Such weak and reversible interactions are highly desirable for practical hydrogen storage applications because they facilitate facile adsorption and desorption under near-ambient conditions.

The hydrogen storage capacity (HSC) of the Li@P–C_3_N system was calculated using eq 6. As summarized in Table 2, the fully saturated Li@P–C_3_N+12H_2_ configuration achieves a maximum gravimetric hydrogen storage capacity of 9.59 wt %, which significantly exceeds the DOE target value of 6.5 wt % for practical hydrogen storage systems. Furthermore, the obtained HSC is comparable to or higher than several previously reported metal-decorated two-dimensional materials, including Na-decorated T-C_3_N (10.63 wt %),^42^ Na-decorated P–C_3_N (9.88 wt %),^65^ NLi_4_-decorated BPB (9.93 wt %),^11^ and Li-decorated BPB (9.05 wt %).^66^ These findings demonstrate that Li@P–C_3_N is a highly promising candidate for reversible hydrogen storage applications. A comparative analysis of adsorption energies, desorption temperatures, and hydrogen storage capacities with previously reported systems is provided in Table 3.

**Table 3:** Comparative Data Analysis of Adsorption Energy (*E*_ads_), Hydrogen Storage Capacity (HSC), and Desorption Temperature (*T_D_*) for Different Hydrogen Storage Materials

System	*E*_ads_ (eV/H_2_)	HSC (wt %)	*T*_*D*_ (K)
NLi_4_@BPB^11^	–0.35/–0.24	9.93	307.04
Li/Na@3D-B_2_P_2_ ^13^	–0.15/–0.10	7.07/6.36	194.32/134.83
Li@DVG^26^	–0.26	7.26	NA
Li@h-B_2_S_3_ ^31^	–0.16	6.35	209.84
Sc/Ti/V@C_3_N_5_ ^33^	–0.27/–0.29/–0.24	9.65/9.48/9.32	NA
Li/Na@IGP-SiC^34^	–0.14/–0.10	8.27/6.78	191.00/148.00
Mg@C_7_N_3_ ^38^	–0.13	6.45	152.00
Li@CN^39^	–0.14	10.81	NA
Li@α-C_3_N_2_ ^40^	–0.22	5.70	285.00
Na@T-C_3_N^42^	–0.11	10.63	140.00
Li@P–C_3_N (Present work)	–0.13	9.59	166.26

### Practical Operation of Li@P–C_3_N for Reversible Hydrogen Storage

According to the DOE targets for onboard hydrogen storage in light-duty fuel-cell vehicles, the practical operating conditions should fall within a temperature range of 233–358 K and a pressure range of 5–12 bar. To evaluate the operational feasibility of the Li@P–C_3_N system under these conditions, the hydrogen desorption temperature (*T*_*D*_) was estimated using the van’t Hoff relation (eq 7), and the calculated values are summarized in Table 2. The predicted desorption temperatures for hydrogen adsorbed on Li@P–C_3_N range from 166.26 to 229.19 K, depending on the adsorption strength. At a reference pressure of 1 atm, the calculated desorption temperatures lie below the near-ambient operating range, indicating that lower temperatures or elevated pressures are required to maintain substantial hydrogen coverage.

To further investigate the pressure dependence of hydrogen desorption, the variation of *T*_*D*_ as a function of pressure was evaluated using the minimum (*E*_ads_^min^), average (*E*_ads_^avg^), and maximum (*E*_ads_^max^) adsorption energies, as presented in Figure 7. In all cases, the desorption temperature increases monotonically with increasing external pressure, demonstrating that the hydrogen adsorption stability can be effectively tuned through pressure modulation. This behavior is highly desirable for practical hydrogen storage systems, as it enables controllable adsorption and desorption processes under realistic operating environments.

**Figure 7:**
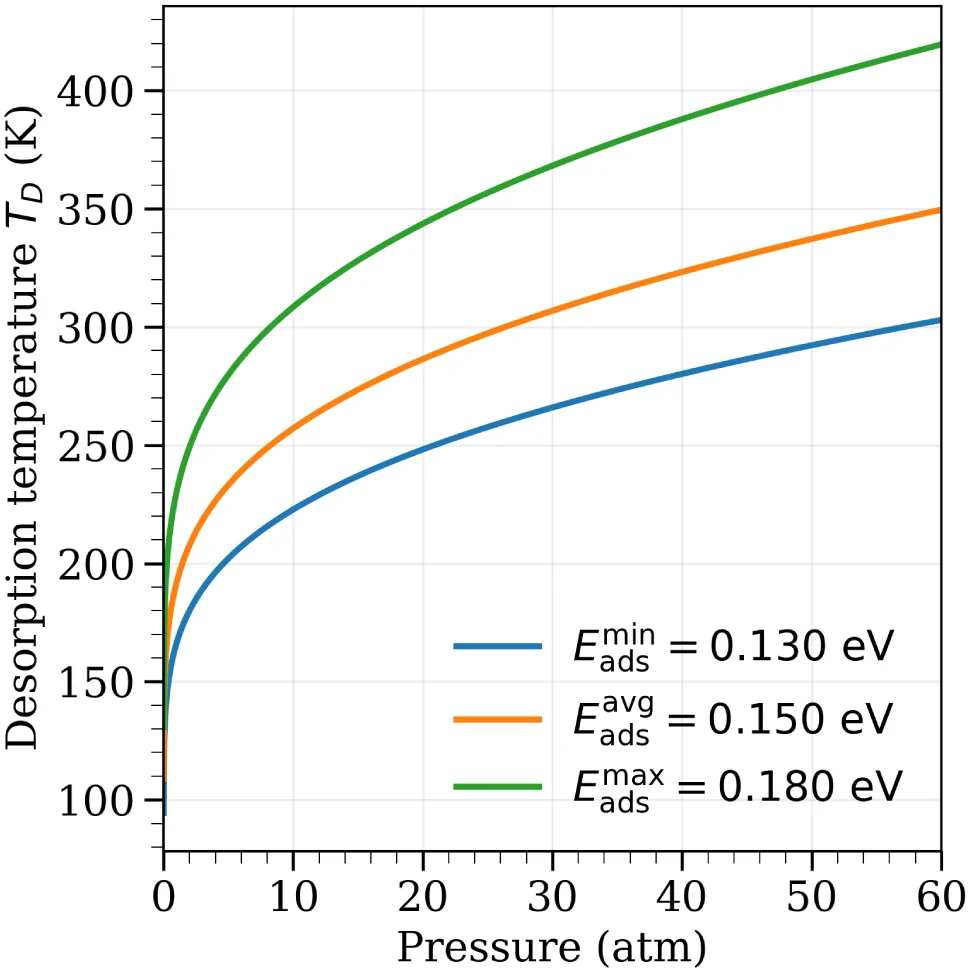
Variation of the hydrogen desorption temperature (*T_D_*) as a function of pressure for the Li@P–C_3_N system, demonstrating the enhancement of desorption temperature with increasing external pressure. The positive values shown correspond to the absolute magnitudes of the calculated adsorption energies.

The practical hydrogen uptake behavior of the Li@P–C_3_N system was further examined by evaluating the hydrogen occupation number (θ = ⟨*N*⟩/*N*_max_) as a function of temperature and pressure using eqs 8–14. The resulting three-dimensional occupation profile is shown in Figure 8a. Adsorption sites remain nearly fully occupied (θ ≈ 1) at low temperatures and elevated pressures, indicating complete adsorption of 12H_2_ molecules on the Li@P–C_3_N surface. As the temperature increases, the hydrogen occupation gradually decreases, reflecting the onset of thermally activated desorption. This behavior confirms that hydrogen adsorption is favored under low-temperature/high-pressure conditions, whereas desorption becomes dominant at elevated temperatures.

**Figure 8:**
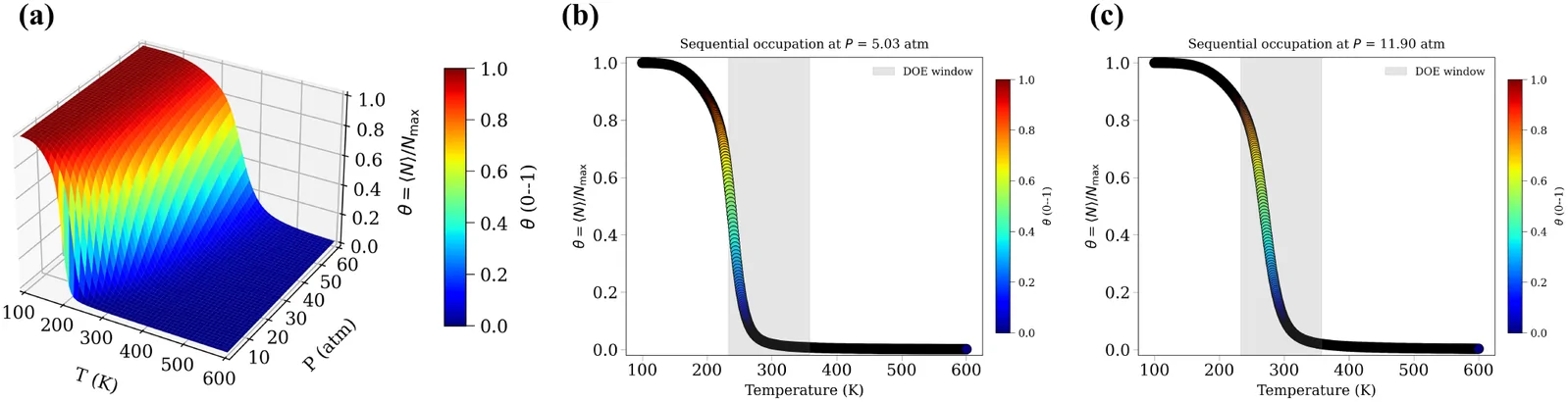
(a) Three-dimensional hydrogen occupation index (θ) showing the variation of hydrogen uptake on the Li-decorated P–C_3_N monolayer (Li@P–C_3_N) as a function of temperature and pressure. The red plateau region (θ ≈ 1) corresponds to fully occupied adsorption sites, whereas the blue region indicates progressive desorption of H_2_ molecules with increasing temperature and/or decreasing pressure. (b, c) Two-dimensional contour projections of θ as a function of temperature at constant pressures of 5.03 and 11.90 atm, respectively. The shaded regions represent the DOE-recommended operating temperature window, highlighting the pressure-dependent adsorption–desorption characteristics of the Li@P–C_3_N system.

To assess the suitability of the system under DOE operating conditions, two-dimensional projections of the occupation profile were extracted at pressures of 5.03 and 11.90 atm, as shown in Figure 8b,c, respectively. The shaded region in the plots represents the DOE-recommended temperature window (233–358 K). At 5.03 atm, the occupation number decreases from approximately θ ≈ 0.5 at 233 K to zero at 358 K, indicating progressive hydrogen desorption within the desired operating range. At a higher pressure of 11.90 atm, the occupation number increases to approximately θ ≈ 0.8 at 233 K, demonstrating enhanced hydrogen retention under elevated pressure conditions. However, upon increasing the temperature to 358 K, the occupation number approaches zero in both cases, indicating complete hydrogen release. These results clearly demonstrate that the adsorption–desorption characteristics of Li@P–C_3_N are strongly pressure dependent and can satisfy the DOE operational requirements for reversible hydrogen storage.

To further examine the thermal stability and reversibility of hydrogen adsorption, *ab initio* molecular dynamics (AIMD) simulations were performed for the fully hydrogen-loaded Li@P–C_3_N+12H_2_ system at 300 K for 20 ps using a time step of 0.5 fs. The final structural configurations obtained after the simulation are presented in Figure 9a,b, corresponding to the top and side views, respectively.

**Figure 9:**
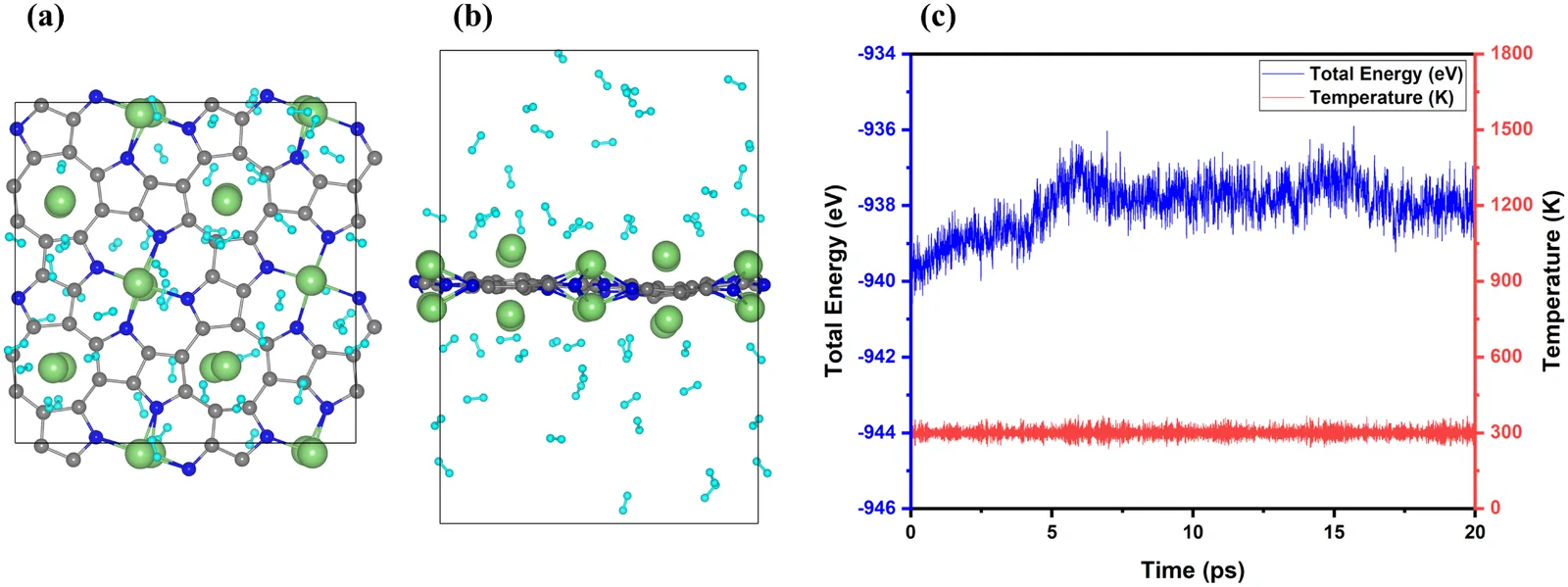
Final AIMD snapshots of Li@P–C_3_N+12H_2_ after 20 ps at 300 K showing the (a) top and (b) side views, illustrating partial desorption of H_2_ molecules. (c) Time evolution of the total energy and temperature during the AIMD simulation performed for 20 ps with a time step of 0.5 fs.

The AIMD results reveal that a fraction of the adsorbed H_2_ molecules spontaneously desorbs from the Li@P–C_3_N surface during the simulation, whereas several hydrogen molecules remain adsorbed on the substrate. This behavior indicates moderate adsorption strength and confirms the reversible adsorption–desorption nature of the system under ambient conditions. Importantly, the structural framework of the Li@P–C_3_N monolayer remains intact throughout the simulation, and the Li adatoms retain their original adsorption positions without noticeable clustering or detachment, demonstrating strong anchoring of Li atoms on the substrate.

The corresponding total energy and temperature profiles are displayed in Figure 9c. The temperature fluctuates narrowly around 300 K, confirming thermal equilibration of the system during the simulation. Although the total energy exhibits moderate fluctuations during the initial equilibration stage, it subsequently stabilizes over the remaining simulation time, indicating good dynamical stability of the hydrogen-loaded structure.

The observed partial yet spontaneous desorption of hydrogen molecules suggests that the interaction between H_2_ and Li@P–C_3_N is sufficiently weak to enable reversible hydrogen release while remaining strong enough to retain a substantial fraction of adsorbed hydrogen at room temperature. These findings collectively demonstrate that Li@P–C_3_N satisfies the essential thermodynamic and kinetic requirements for practical reversible hydrogen storage and may serve as a promising candidate for onboard fuel-cell vehicle applications.

## Conclusions

In summary, first-principles calculations demonstrate that Li-decorated P–C_3_N (Li@P–C_3_N) is a promising candidate for reversible hydrogen storage applications. The calculated Li binding energy, which exceeds the cohesive energy of bulk Li, demonstrates strong anchoring of Li adatoms on the P–C_3_N surface and effectively suppresses metal aggregation. The Li-decorated system can accommodate up to 12H_2_ molecules with adsorption energies ranging from −0.13 to −0.18 eV per H_2_, which lies within the desirable thermodynamic window for reversible hydrogen adsorption and desorption. The corresponding gravimetric hydrogen storage capacity reaches 9.59 wt %, exceeding the U.S. Department of Energy (DOE) target for practical hydrogen storage systems.

The strong stability of Li decoration is further supported by the high Li diffusion barriers of 3.08 and 3.11 eV, indicating negligible Li migration and a low probability of metal clustering on the P–C_3_N surface. Electronic structure analyses, including charge density difference, Bader charge, and projected density of states (PDOS), reveal significant charge transfer from Li atoms to the P–C_3_N monolayer, leading to polarization of the adsorbed H_2_ molecules and enhanced adsorption capability. Moreover, noncovalent interaction (NCI) analysis confirms that hydrogen adsorption is predominantly governed by weak van der Waals interactions, which are essential for reversible hydrogen storage.

Thermal and dynamical stability of the Li@P–C_3_N system are validated through *ab initio* molecular dynamics (AIMD) simulations and phonon dispersion calculations, respectively. The AIMD results demonstrate structural integrity and reversible adsorption–desorption behavior under ambient conditions, while the absence of imaginary phonon frequencies confirms the dynamical stability of the Li-decorated monolayer. Thus, these findings establish Li@P–C_3_N as a highly stable and efficient hydrogen storage material with strong potential for practical reversible hydrogen storage applications.

## Supplementary Material



## Data Availability

Data supporting the results can be accessed by contacting the corresponding author.

## Notes

Final structural snapshot obtained from the AIMD simulation of pristine P–C_3_N, optimized adsorption geometry of H_2_ molecules at various possible adsorption sites on the pristine P–C_3_N monolayer, and final structural snapshot from the AIMD simulation of the Li-decorated P–C_3_N (Li@P–C_3_N) system (PDF)
