# Supplementary material for: Impact of Li Decoration on P‑C3N Monolayer for Reversible Hydrogen Storage: A First-Principles Study
Source: Langmuir. 2026 Aug 6;42(32):23642–55. doi: 10.1021/acs.langmuir.6c03071 (PMC13492364; doi:10.1021/acs.langmuir.6c03071)
Supplement: Supplementary file 1 [file la6c03071_si_001.pdf]

Supporting Information

Impact of Li Decoration on P-C_3N Monolayer for Reversible Hydrogen Storage: A First-Principles Study

Amit Ramchiary[1], José A. S. Laranjeira[2,*], Luiz A. Ribeiro Jr.[3,4], Julio R. Sambrano[2], and Paritosh Mondal[1,*]

[1]Department of Chemistry, Assam University, Silchar 788011, Assam, India
[2]Modeling and Molecular Simulation Group, São Paulo State University (UNESP), School of Sciences, Bauru 17033-360, SP, Brazil
[3]Institute of Physics, University of Brasília, Brasília 70910-900, DF, Brazil
[4]Computational Materials Laboratory (LCCMat), Institute of Physics, University of Brasília, Brasília 70910-900, DF, Brazil

***Corresponding Authors**
E-mail: jose.laranjeira@unesp.br; paritos_au@yahoo.co.in

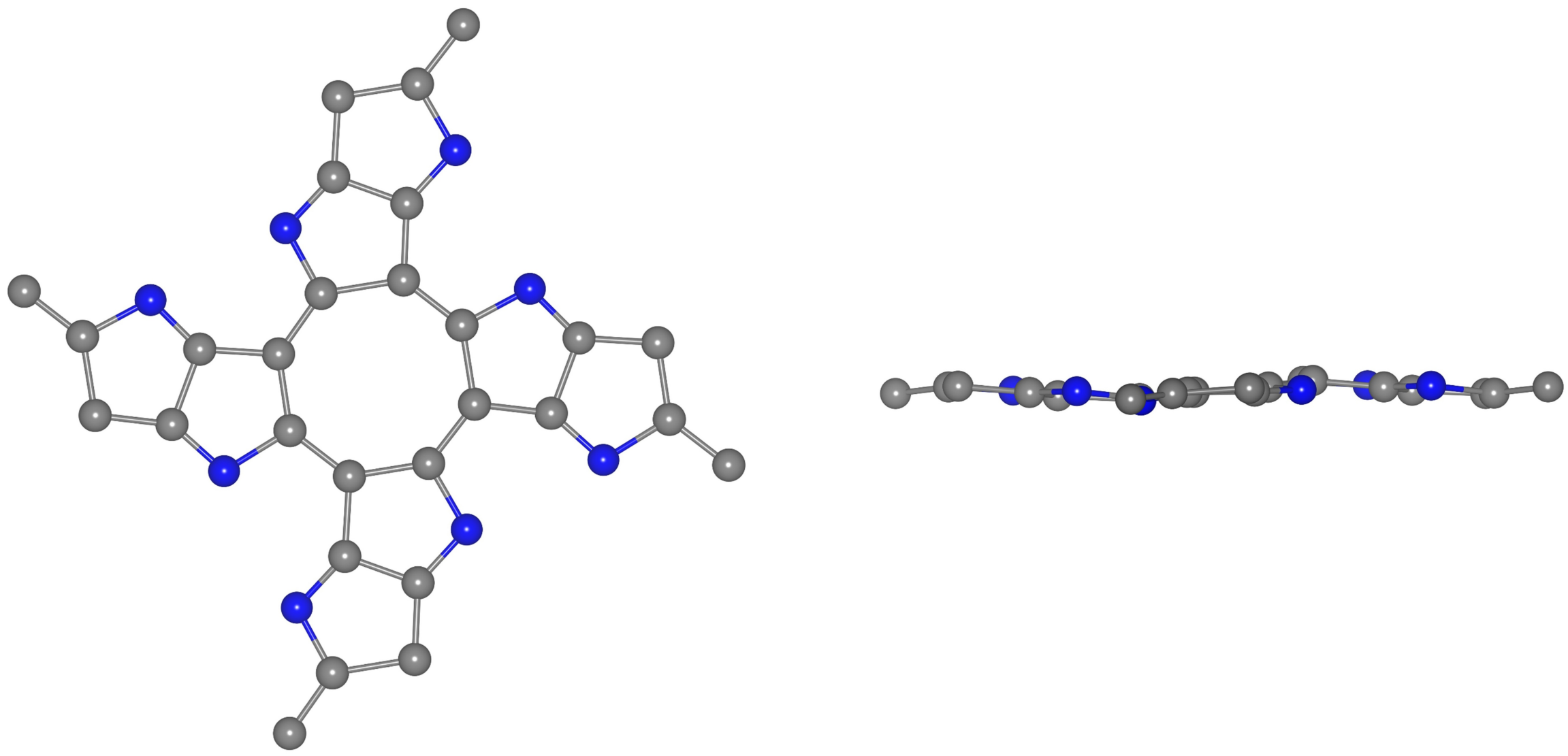

Figure S1. Top and side views of the AIMD simulation snapshot of pristine P-C_3N at 300 K after 5 ps.

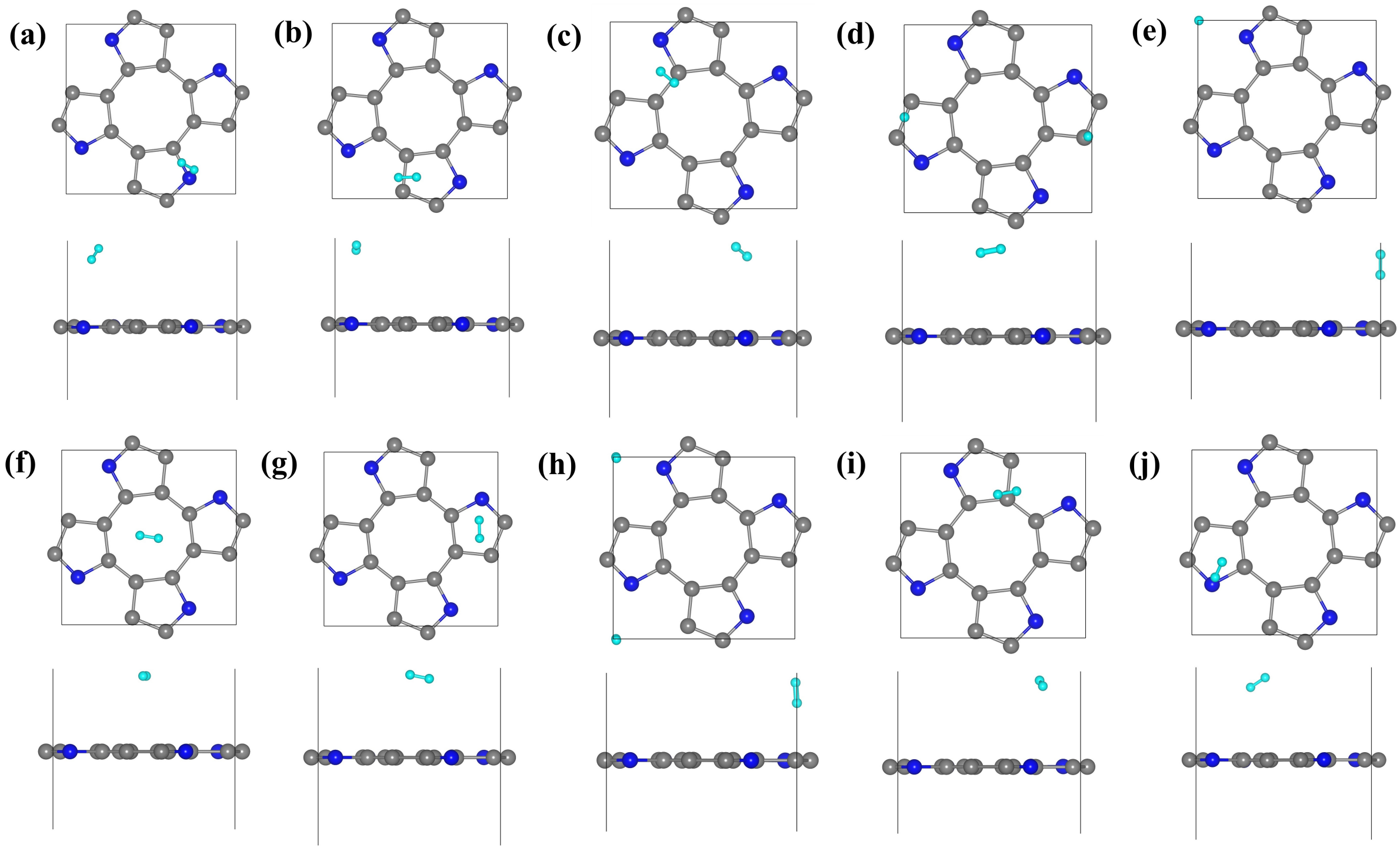

Figure S2. Top and side views of the optimized structures for the adsorption of a single H_2 molecule on the pristine P-C_3N monolayer at different adsorption sites: (a) B1, (b) B2, (c) B3, (d) B4, (e) H1, (f) H2, (g) H3, (h) T1, (i) T2, and (j) T3. Hydrogen atoms are shown in cyan.

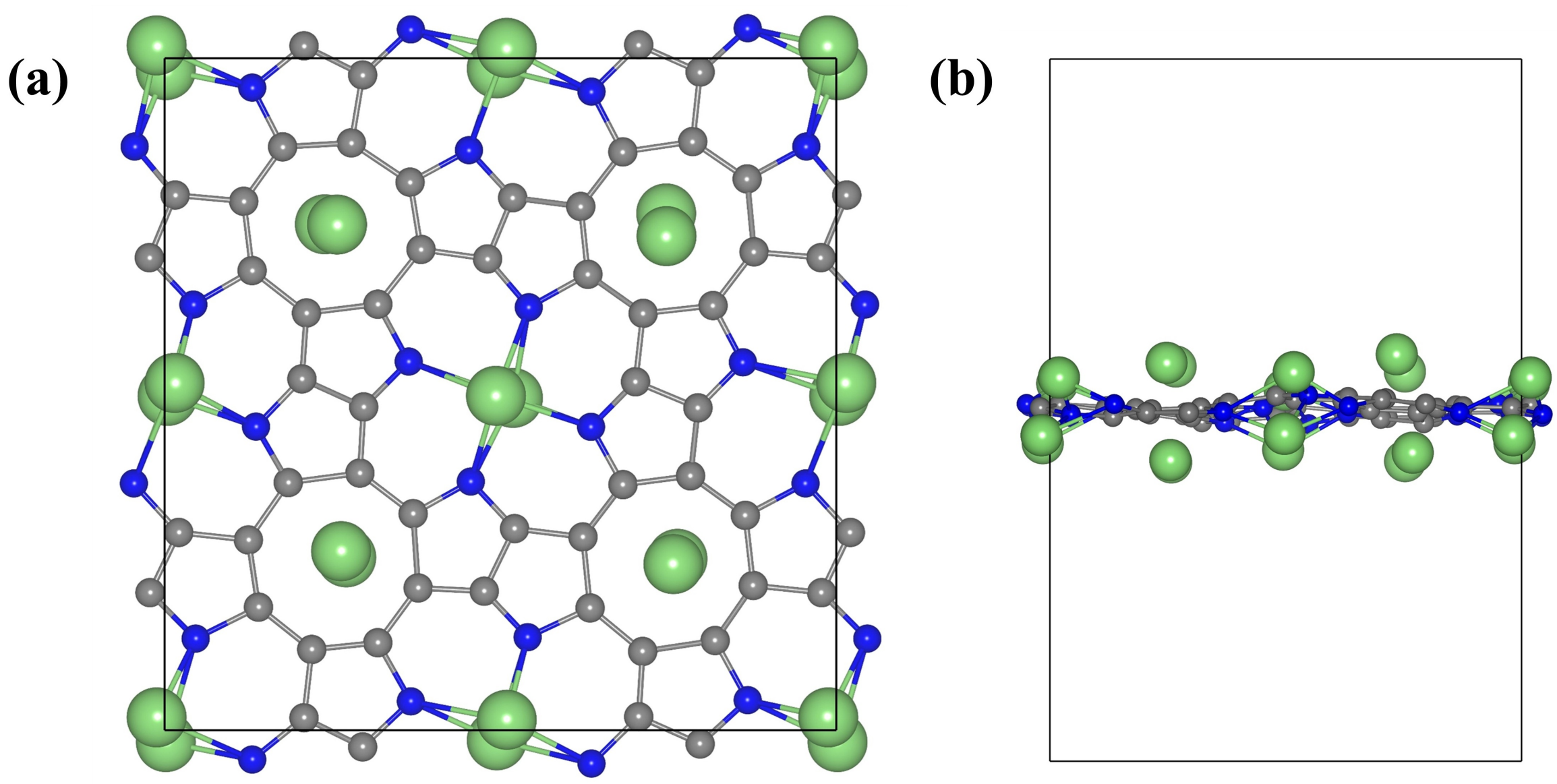

Figure S3. (a) Top and (b) front views of the AIMD simulation snapshot of Li@P-C3N at 450 K for 20 ps.
